# Methyl 2-diphenyl­phosphor­yloxy-2-aza­bicyclo­[2.2.1]hept-5-ene-3-*exo*-carboxyl­ate

**DOI:** 10.1107/S160053680804292X

**Published:** 2008-12-20

**Authors:** Carlos A. D. Sousa, M. Luísa C. Vale, José E. Rodríguez-Borges, Xerardo Garcia-Mera

**Affiliations:** a Centro de Investigação em Química, Departamento de Química, Faculdade de Ciências, Universidade do Porto, Rua do Campo Alegre, 687 4169-007, Porto, Portugal; b Departamento de Química Orgánica, Facultade de Farmacia, Universidade de Santiago de Compostela, E-15782 Santiago de Compostela, Spain

## Abstract

In the title compound, C_20_H_20_NO_4_P, the dihedral angle between the phenyl rings is 68.52 (7)°. In the crystal structure, the mol­ecules are linked by a weak C—H⋯π(arene) inter­action along [010] involving the phenyl CH group and the phenyl rings. There are no further significant inter­molecular inter­actions.

## Related literature

For the preparation of the precursor of the title compound, see: Sousa *et al*. (2008 ▶). For related literature about this type of bicyclic compound and their relevance see: Vale *et al.* (2006 ▶), Alves *et al.* (2006 ▶), Yoda *et al.* (1995 ▶).
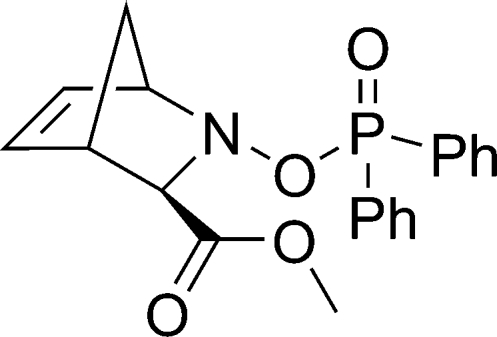


## Experimental

### 

#### Crystal data

C_20_H_20_NO_4_P
*M* *_r_* = 369.34
Monoclinic, 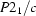

*a* = 18.4223 (6) Å
*b* = 8.5522 (3) Å
*c* = 11.6022 (4) Å
β = 97.1810 (10)°
*V* = 1813.60 (11) Å^3^
*Z* = 4
Mo *K*α radiation
μ = 0.18 mm^−1^
*T* = 100 (2) K
0.37 × 0.34 × 0.34 mm

#### Data collection

Bruker APEXII CCD area-detector diffractometer
Absorption correction: multi-scan (*SADABS*; Bruker, 2006 ▶) *T*_min_ = 0.871, *T*_max_ = 0.940
14828 measured reflections
3717 independent reflections
3172 reflections with *I* > 2σ(*I*)
*R*_int_ = 0.033

#### Refinement

*R*[*F* ^2^ > 2σ(*F* ^2^)] = 0.035
*wR*(*F* ^2^) = 0.091
*S* = 1.05
3717 reflections
236 parameters
H-atom parameters constrained
Δρ_max_ = 0.29 e Å^−3^
Δρ_min_ = −0.41 e Å^−3^

### 

Data collection: *APEX2* (Bruker, 2006 ▶); cell refinement: *SAINT* (Bruker, 2006 ▶); data reduction: *SAINT*; program(s) used to solve structure: *SIR97* (Altomare *et al.*, 1997 ▶); program(s) used to refine structure: *SHELXL97* (Sheldrick, 2008 ▶); molecular graphics: *ORTEP-3 for Windows* (Farrugia, 1997 ▶) and *PLATON* (Spek, 2003 ▶); software used to prepare material for publication: *WinGX* publication routines (Farrugia, 1999 ▶).

## Supplementary Material

Crystal structure: contains datablocks I, New_Global_Publ_Block. DOI: 10.1107/S160053680804292X/bx2188sup1.cif

Structure factors: contains datablocks I. DOI: 10.1107/S160053680804292X/bx2188Isup2.hkl

Additional supplementary materials:  crystallographic information; 3D view; checkCIF report

## Figures and Tables

**Table 1:** Hydrogen-bond geometry (Å, °)

*D*—H⋯*A*	*D*—H	H⋯*A*	*D*⋯*A*	*D*—H⋯*A*
C12—H12⋯*Cg*1^i^	0.95	2.77	3.566 (2)	142

Symmetry code: (i) 
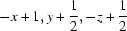
. *Cg*1 is the centroid of the C15–C20 ring.

**Fig. 1.**
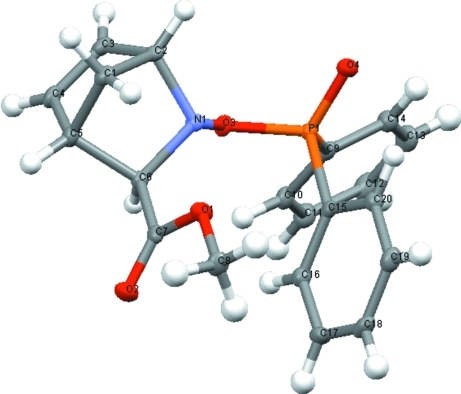
A view of (I), showing the three chiral carbons C2, C5 and C6 and the atom-labelling scheme. Displacement ellipsoids are drawn at the 50% probability level. H atoms are shown as spheres of arbitary radius.

**Fig. 2.**
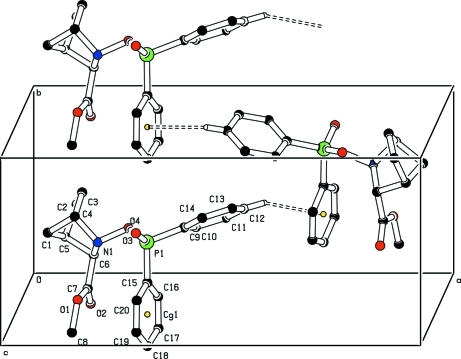
Part of the crystal structute of (I) viewed along the c axis. Dashed lines show C—H···π (arene) interactions. Only H atoms participating in hydrogen bonding are shown. π is the centroid of the ring defined by atoms C15-C20.

## supplementary crystallographic information

### Comment

The stucture of the title compound, (I), is shown in Fig. 1. It can be seen the existence of three chiral centers at C2 (*R*), C5 (S) and C6 (*R*). In the crystalline structure, the molecules are linked by a weak C—H···π interaction, Fig. 2 [H12-π^i^ 2.77 Å, C12-H12-π 142°, C12-π 3.566 (2) Å, symmetry code: (i) 1-x,1/2+y, 1/2-z] along [010] directions. There are no further significant intermolecular interactions.

### Experimental

The title compound was synthesized from the previously prepared (3*exo*)-2-hydroxy-2-azabicyclo[2.2.1]hept-5-ene-3-carboxylate (Sousa *et al.* 2008). Equimolar amounts of (3*exo*)-2-hydroxy-2-azabicyclo[2.2.1]hept-5-ene-3-carboxylate (0.56 g, 3.3 mmol) and diphenylpfosphinic chloride (0.63 ml, 3.3 mmol), in the presence of 1 eq. of anidrous triethylamine and and a catalytic quantity of DMAP, were let to react overnigth in dichloromethane, at room temperature under argon atmosphere. Water was added and the product was extracted with dichloromethane (3 × 15 ml). The organic layers were dried over sodium sulfate and the solvent was evaporated. The obtained product was purified by flash chromatography (eluent: dichloromethane/diethyl ether 1:1), leading to a light clear yellow oil in 80% yield. Crystals of (I) were made from a slow evaporation of a dichloromethane/hexane solution.

### Refinement

All H atoms were found in a difference Fourier map and placed in geometrically idealized and constrained to ride on their parent atoms [C—H = 0.95–1.00 Å and U_iso_(H) = 1.2 or 1.5U_eq_(C)].

### Crystal data

**Table:**

C_20_H_20_NO_4_P	*F*(000) = 776
*M**_r_* = 369.34	*D*_x_ = 1.353 Mg m^−^^3^
Monoclinic, *P*2_1_/*c*	Mo *K*α radiation, λ = 0.71073 Å
Hall symbol: -P 2ybc	Cell parameters from 1953 reflections
*a* = 18.4223 (6) Å	θ = 3.1–25.9°
*b* = 8.5522 (3) Å	µ = 0.18 mm^−^^1^
*c* = 11.6022 (4) Å	*T* = 100 K
β = 97.181 (1)°	Prism, colourless
*V* = 1813.60 (11) Å^3^	0.37 × 0.34 × 0.34 mm
*Z* = 4	

### Data collection

**Table:**

Bruker ApexII CCD area-detector diffractometer	3717 independent reflections
Radiation source: sealed tube	3172 reflections with *I* > 2σ(*I*)
graphite	*R*_int_ = 0.033
phi and ω scans	θ_max_ = 26.4°, θ_min_ = 2.2°
Absorption correction: multi-scan (*SADABS*; Bruker, 2006)	*h* = −23→22
*T*_min_ = 0.871, *T*_max_ = 0.940	*k* = 0→10
14828 measured reflections	*l* = 0→14

### Refinement

**Table:**

Refinement on *F*^2^	Primary atom site location: structure-invariant direct methods
Least-squares matrix: full	Secondary atom site location: difference Fourier map
*R*[*F*^2^ > 2σ(*F*^2^)] = 0.035	Hydrogen site location: inferred from neighbouring sites
*wR*(*F*^2^) = 0.091	H-atom parameters constrained
*S* = 1.05	*w* = 1/[σ^2^(*F*_o_^2^) + (0.0429*P*)^2^ + 0.8843*P*] where *P* = (*F*_o_^2^ + 2*F*_c_^2^)/3
3717 reflections	(Δ/σ)_max_ = 0.001
236 parameters	Δρ_max_ = 0.28 e Å^−^^3^
0 restraints	Δρ_min_ = −0.41 e Å^−^^3^

### Special details

**Table:**

Geometry. All e.s.d.'s (except the e.s.d. in the dihedral angle between two l.s. planes) are estimated using the full covariance matrix. The cell e.s.d.'s are taken into account individually in the estimation of e.s.d.'s in distances, angles and torsion angles; correlations between e.s.d.'s in cell parameters are only used when they are defined by crystal symmetry. An approximate (isotropic) treatment of cell e.s.d.'s is used for estimating e.s.d.'s involving l.s. planes.
Refinement. Refinement of *F*^2^ against ALL reflections. The weighted *R*-factor *wR* and goodness of fit *S* are based on *F*^2^, conventional *R*-factors *R* are based on *F*, with *F* set to zero for negative *F*^2^. The threshold expression of *F*^2^ > σ(*F*^2^) is used only for calculating *R*-factors(gt) *etc*. and is not relevant to the choice of reflections for refinement. *R*-factors based on *F*^2^ are statistically about twice as large as those based on *F*, and *R*- factors based on ALL data will be even larger.

### Fractional atomic coordinates and isotropic or equivalent isotropic displacement parameters (Å^2^)

**Table:**

	*x*	*y*	*z*	*U*_iso_*/*U*_eq_	
C1	0.04005 (8)	0.24766 (17)	0.08174 (13)	0.0156 (3)	
H1A	−0.005	0.3099	0.0614	0.019*	
H1B	0.0318	0.1646	0.1381	0.019*	
C2	0.10739 (8)	0.34831 (17)	0.12086 (13)	0.0140 (3)	
H2	0.1038	0.4159	0.1902	0.017*	
C3	0.11615 (8)	0.43459 (18)	0.00937 (13)	0.0157 (3)	
H3	0.1328	0.539	0.003	0.019*	
C4	0.09614 (8)	0.33679 (18)	−0.07788 (13)	0.0170 (3)	
H4	0.0965	0.358	−0.1582	0.02*	
C5	0.07271 (8)	0.18529 (18)	−0.02579 (13)	0.0152 (3)	
H5	0.0408	0.1144	−0.0788	0.018*	
C6	0.14566 (8)	0.11298 (17)	0.03659 (12)	0.0124 (3)	
H6	0.1849	0.1189	−0.0155	0.015*	
C7	0.13407 (7)	−0.05517 (17)	0.07081 (12)	0.0126 (3)	
C8	0.10791 (9)	−0.23359 (18)	0.21479 (14)	0.0188 (3)	
H8A	0.0638	−0.2759	0.1694	0.028*	
H8B	0.1021	−0.2364	0.2976	0.028*	
H8C	0.1503	−0.2968	0.201	0.028*	
C9	0.37374 (8)	0.32042 (17)	0.19955 (13)	0.0135 (3)	
C10	0.39313 (8)	0.28107 (18)	0.09030 (13)	0.0156 (3)	
H10	0.3592	0.2281	0.0353	0.019*	
C11	0.46227 (8)	0.31986 (19)	0.06275 (14)	0.0196 (3)	
H11	0.4758	0.2923	−0.011	0.024*	
C12	0.51155 (8)	0.3985 (2)	0.14240 (15)	0.0220 (4)	
H12	0.559	0.4232	0.1236	0.026*	
C13	0.49180 (9)	0.4412 (2)	0.24924 (15)	0.0228 (4)	
H13	0.5253	0.4974	0.3029	0.027*	
C14	0.42326 (8)	0.40209 (18)	0.27826 (14)	0.0180 (3)	
H14	0.41	0.431	0.3519	0.022*	
C15	0.29184 (7)	0.05353 (17)	0.27096 (13)	0.0125 (3)	
C16	0.31090 (8)	−0.05303 (18)	0.18853 (13)	0.0156 (3)	
H16	0.3218	−0.017	0.1151	0.019*	
C17	0.31393 (9)	−0.21192 (18)	0.21417 (14)	0.0190 (3)	
H17	0.3284	−0.2841	0.1591	0.023*	
C18	0.29590 (8)	−0.26536 (18)	0.31971 (15)	0.0200 (3)	
H18	0.2972	−0.3742	0.3364	0.024*	
C19	0.27594 (8)	−0.16006 (19)	0.40103 (14)	0.0185 (3)	
H19	0.2629	−0.197	0.4729	0.022*	
C20	0.27503 (8)	−0.00078 (18)	0.37771 (13)	0.0149 (3)	
H20	0.2629	0.0713	0.4346	0.018*	
N1	0.16332 (6)	0.21932 (14)	0.13812 (11)	0.0116 (3)	
O1	0.11889 (6)	−0.07336 (12)	0.17981 (9)	0.0166 (2)	
O2	0.13582 (6)	−0.16137 (12)	0.00278 (9)	0.0190 (2)	
O3	0.23563 (5)	0.28855 (12)	0.12671 (9)	0.0131 (2)	
O4	0.27071 (6)	0.34776 (12)	0.34900 (9)	0.0158 (2)	
P1	0.288697 (19)	0.26111 (4)	0.24650 (3)	0.01112 (11)	

### Atomic displacement parameters (Å^2^)

**Table:**

	*U*^11^	*U*^22^	*U*^33^	*U*^12^	*U*^13^	*U*^23^
C1	0.0105 (7)	0.0152 (8)	0.0210 (8)	0.0011 (6)	0.0016 (6)	0.0022 (6)
C2	0.0121 (7)	0.0126 (7)	0.0175 (7)	0.0026 (6)	0.0025 (6)	−0.0004 (6)
C3	0.0118 (7)	0.0132 (7)	0.0218 (8)	0.0026 (6)	0.0016 (6)	0.0041 (6)
C4	0.0154 (7)	0.0179 (8)	0.0172 (7)	0.0029 (6)	0.0000 (6)	0.0060 (6)
C5	0.0130 (7)	0.0149 (8)	0.0167 (7)	−0.0011 (6)	−0.0020 (6)	0.0021 (6)
C6	0.0112 (7)	0.0123 (7)	0.0135 (7)	−0.0005 (6)	0.0008 (5)	0.0000 (6)
C7	0.0081 (6)	0.0149 (7)	0.0145 (7)	0.0003 (6)	−0.0001 (5)	−0.0004 (6)
C8	0.0206 (8)	0.0145 (8)	0.0209 (8)	−0.0045 (6)	0.0010 (6)	0.0058 (6)
C9	0.0106 (7)	0.0116 (7)	0.0180 (7)	0.0001 (6)	0.0005 (6)	0.0035 (6)
C10	0.0133 (7)	0.0161 (8)	0.0169 (7)	−0.0018 (6)	−0.0003 (6)	0.0041 (6)
C11	0.0170 (8)	0.0232 (8)	0.0192 (8)	0.0001 (6)	0.0047 (6)	0.0046 (6)
C12	0.0131 (7)	0.0242 (9)	0.0289 (9)	−0.0026 (6)	0.0031 (6)	0.0075 (7)
C13	0.0152 (8)	0.0235 (9)	0.0283 (9)	−0.0056 (7)	−0.0031 (7)	−0.0009 (7)
C14	0.0157 (7)	0.0174 (8)	0.0205 (8)	−0.0009 (6)	0.0002 (6)	−0.0009 (6)
C15	0.0086 (7)	0.0114 (7)	0.0165 (7)	−0.0001 (5)	−0.0021 (5)	0.0011 (6)
C16	0.0139 (7)	0.0163 (8)	0.0162 (7)	0.0004 (6)	0.0008 (6)	0.0004 (6)
C17	0.0188 (8)	0.0145 (8)	0.0226 (8)	0.0031 (6)	−0.0022 (6)	−0.0060 (6)
C18	0.0166 (8)	0.0120 (8)	0.0296 (9)	−0.0001 (6)	−0.0043 (7)	0.0042 (7)
C19	0.0151 (7)	0.0198 (8)	0.0202 (8)	−0.0022 (6)	0.0002 (6)	0.0068 (6)
C20	0.0121 (7)	0.0154 (8)	0.0169 (7)	−0.0008 (6)	0.0008 (6)	−0.0007 (6)
N1	0.0071 (6)	0.0117 (6)	0.0161 (6)	−0.0022 (5)	0.0017 (5)	−0.0004 (5)
O1	0.0224 (6)	0.0119 (5)	0.0163 (5)	−0.0032 (4)	0.0049 (4)	0.0011 (4)
O2	0.0245 (6)	0.0139 (6)	0.0189 (6)	−0.0012 (4)	0.0038 (5)	−0.0031 (5)
O3	0.0079 (5)	0.0142 (5)	0.0168 (5)	−0.0033 (4)	0.0003 (4)	0.0029 (4)
O4	0.0165 (5)	0.0135 (5)	0.0177 (5)	−0.0003 (4)	0.0030 (4)	−0.0014 (4)
P1	0.00981 (19)	0.01024 (19)	0.0131 (2)	−0.00066 (14)	0.00083 (14)	0.00053 (14)

### Geometric parameters (Å, °)

**Table:**

C1—C2	1.531 (2)	C10—C11	1.392 (2)
C1—C5	1.546 (2)	C10—H10	0.95
C1—H1A	0.99	C11—C12	1.386 (2)
C1—H1B	0.99	C11—H11	0.95
C2—N1	1.5057 (18)	C12—C13	1.384 (2)
C2—C3	1.515 (2)	C12—H12	0.95
C2—H2	1	C13—C14	1.388 (2)
C3—C4	1.329 (2)	C13—H13	0.95
C3—H3	0.95	C14—H14	0.95
C4—C5	1.515 (2)	C15—C20	1.393 (2)
C4—H4	0.95	C15—C16	1.397 (2)
C5—C6	1.571 (2)	C15—P1	1.7976 (15)
C5—H5	1	C16—C17	1.391 (2)
C6—N1	1.4915 (18)	C16—H16	0.95
C6—C7	1.514 (2)	C17—C18	1.386 (2)
C6—H6	1	C17—H17	0.95
C7—O2	1.2064 (18)	C18—C19	1.387 (2)
C7—O1	1.3379 (17)	C18—H18	0.95
C8—O1	1.4505 (18)	C19—C20	1.389 (2)
C8—H8A	0.98	C19—H19	0.95
C8—H8B	0.98	C20—H20	0.95
C8—H8C	0.98	N1—O3	1.4786 (15)
C9—C14	1.395 (2)	O3—P1	1.6133 (10)
C9—C10	1.400 (2)	O4—P1	1.4737 (11)
C9—P1	1.7950 (15)		
			
C2—C1—C5	92.89 (11)	C11—C10—H10	120.2
C2—C1—H1A	113.1	C9—C10—H10	120.2
C5—C1—H1A	113.1	C12—C11—C10	120.30 (15)
C2—C1—H1B	113.1	C12—C11—H11	119.8
C5—C1—H1B	113.1	C10—C11—H11	119.8
H1A—C1—H1B	110.5	C13—C12—C11	120.11 (14)
N1—C2—C3	109.04 (11)	C13—C12—H12	119.9
N1—C2—C1	98.23 (11)	C11—C12—H12	119.9
C3—C2—C1	100.92 (12)	C12—C13—C14	120.20 (15)
N1—C2—H2	115.5	C12—C13—H13	119.9
C3—C2—H2	115.5	C14—C13—H13	119.9
C1—C2—H2	115.5	C13—C14—C9	120.14 (15)
C4—C3—C2	107.14 (13)	C13—C14—H14	119.9
C4—C3—H3	126.4	C9—C14—H14	119.9
C2—C3—H3	126.4	C20—C15—C16	119.59 (14)
C3—C4—C5	107.46 (13)	C20—C15—P1	117.59 (11)
C3—C4—H4	126.3	C16—C15—P1	122.82 (11)
C5—C4—H4	126.3	C17—C16—C15	119.85 (14)
C4—C5—C1	100.71 (12)	C17—C16—H16	120.1
C4—C5—C6	104.50 (12)	C15—C16—H16	120.1
C1—C5—C6	99.25 (11)	C18—C17—C16	120.24 (15)
C4—C5—H5	116.6	C18—C17—H17	119.9
C1—C5—H5	116.6	C16—C17—H17	119.9
C6—C5—H5	116.6	C17—C18—C19	120.02 (14)
N1—C6—C7	113.33 (11)	C17—C18—H18	120
N1—C6—C5	102.32 (11)	C19—C18—H18	120
C7—C6—C5	110.74 (12)	C18—C19—C20	120.14 (15)
N1—C6—H6	110.1	C18—C19—H19	119.9
C7—C6—H6	110.1	C20—C19—H19	119.9
C5—C6—H6	110.1	C19—C20—C15	120.11 (14)
O2—C7—O1	123.83 (14)	C19—C20—H20	119.9
O2—C7—C6	121.82 (13)	C15—C20—H20	119.9
O1—C7—C6	114.29 (12)	O3—N1—C6	106.47 (10)
O1—C8—H8A	109.5	O3—N1—C2	107.69 (10)
O1—C8—H8B	109.5	C6—N1—C2	105.28 (11)
H8A—C8—H8B	109.5	C7—O1—C8	115.30 (12)
O1—C8—H8C	109.5	N1—O3—P1	108.68 (8)
H8A—C8—H8C	109.5	O4—P1—O3	116.67 (6)
H8B—C8—H8C	109.5	O4—P1—C9	113.30 (7)
C14—C9—C10	119.54 (13)	O3—P1—C9	98.94 (6)
C14—C9—P1	117.84 (12)	O4—P1—C15	112.04 (7)
C10—C9—P1	122.54 (11)	O3—P1—C15	106.49 (6)
C11—C10—C9	119.67 (14)	C9—P1—C15	108.35 (7)
			
C5—C1—C2—N1	−60.85 (12)	C18—C19—C20—C15	2.1 (2)
C5—C1—C2—C3	50.47 (12)	C16—C15—C20—C19	−1.2 (2)
N1—C2—C3—C4	68.17 (15)	P1—C15—C20—C19	179.43 (11)
C1—C2—C3—C4	−34.58 (15)	C7—C6—N1—O3	120.70 (12)
C2—C3—C4—C5	0.88 (16)	C5—C6—N1—O3	−120.03 (11)
C3—C4—C5—C1	32.73 (15)	C7—C6—N1—C2	−125.14 (12)
C3—C4—C5—C6	−69.86 (15)	C5—C6—N1—C2	−5.87 (13)
C2—C1—C5—C4	−49.87 (12)	C3—C2—N1—O3	51.25 (14)
C2—C1—C5—C6	56.93 (12)	C1—C2—N1—O3	155.87 (10)
C4—C5—C6—N1	71.20 (13)	C3—C2—N1—C6	−62.06 (14)
C1—C5—C6—N1	−32.48 (13)	C1—C2—N1—C6	42.56 (13)
C4—C5—C6—C7	−167.72 (12)	O2—C7—O1—C8	3.0 (2)
C1—C5—C6—C7	88.59 (13)	C6—C7—O1—C8	−179.72 (12)
N1—C6—C7—O2	−162.95 (13)	C6—N1—O3—P1	−127.24 (9)
C5—C6—C7—O2	82.74 (17)	C2—N1—O3—P1	120.25 (10)
N1—C6—C7—O1	19.76 (17)	N1—O3—P1—O4	−68.47 (10)
C5—C6—C7—O1	−94.56 (14)	N1—O3—P1—C9	169.71 (9)
C14—C9—C10—C11	1.8 (2)	N1—O3—P1—C15	57.44 (9)
P1—C9—C10—C11	−174.85 (12)	C14—C9—P1—O4	18.47 (14)
C9—C10—C11—C12	−0.7 (2)	C10—C9—P1—O4	−164.79 (12)
C10—C11—C12—C13	−1.0 (2)	C14—C9—P1—O3	142.70 (12)
C11—C12—C13—C14	1.6 (3)	C10—C9—P1—O3	−40.56 (14)
C12—C13—C14—C9	−0.4 (2)	C14—C9—P1—C15	−106.51 (13)
C10—C9—C14—C13	−1.3 (2)	C10—C9—P1—C15	70.23 (14)
P1—C9—C14—C13	175.54 (12)	C20—C15—P1—O4	2.24 (13)
C20—C15—C16—C17	−0.9 (2)	C16—C15—P1—O4	−177.14 (11)
P1—C15—C16—C17	178.48 (11)	C20—C15—P1—O3	−126.43 (11)
C15—C16—C17—C18	2.0 (2)	C16—C15—P1—O3	54.19 (13)
C16—C17—C18—C19	−1.1 (2)	C20—C15—P1—C9	127.97 (11)
C17—C18—C19—C20	−1.0 (2)	C16—C15—P1—C9	−51.41 (14)

### Hydrogen-bond geometry (Å, °)

**Table:**

*D*—H···*A*	*D*—H	H···*A*	*D*···*A*	*D*—H···*A*
C12—H12···Cg1^i^	0.95	2.77	3.566 (2)	142

Symmetry codes: (i) −*x*+1, *y*+1/2, −*z*+1/2.
